# Supplementary material for: Hallmarks of primary neurulation are conserved in the zebrafish forebrain
Source: Commun Biol. 2021 Jan 29;4:147. doi: 10.1038/s42003-021-01655-8 (PMC7846805; doi:10.1038/s42003-021-01655-8)
Supplement: Supplementary file 2 — Description of Additional Supplementary Files [file 42003_2021_1655_MOESM2_ESM.pdf]

Description of Additional Supplementary Files

File name: Supplemental Movie 1.

Description: 3-Dimensional rotation revealing organization of neural fold cells in a 5 som embryo. Transverse section, at the level of the forebrain of a 5 somite embryo mosaically-expressing mGFP.

File name: Supplemental Movie 2.

Description: 3-Dimensional rotation revealing organization of neural fold cells in a 7 som embryo. Transverse section, at the level of the forebrain of a 7 somite embryo mosaically-expressing mGFP.

File name: Supplemental Movie 3.

Description: MHP cells become incorporated into the deep layer of the eye field. Time lapse imaging of embryo ubiquitously expressing mKaede (green) that was photo-converted in medial superficial cells that form the MHP (magenta). MHP cells sink inwards and become incorporated into the deep layer of the eye field as neural folds elevate. Annotations: white arrows: sinking of MHP cells.

File name: Supplemental Movie 4.

Description: Neural fold cells constrict basally as neural folds elevate. Time lapse imaging of Tg[emx3:YFP] transgenic embryo ubiquitously expressing mRFP. YFP-positive neural fold cells (green) constrict basally as neural folds elevate. Annotations: Cyan arrow: basal surface of neural fold cells; white lines: telencephalon cells expressing YFP.

File name: Supplemental Movie 5.

Description: MHP cells undergo oscillatory constriction with decreasing amplitude. Time lapse imaging of embryo ubiquitously expressing mGFP. Clusters of medial (MHP) constrict apically in an oscillatory manner, in contrast to their lateral neighbors that do not. Annotations: cyan dots = MHP cells; yellow asterisks = MHP-adjacent cells that do not undergo apical constriction, yellow dashed line: midline, red asterisks = EVL cells.

File name: Supplemental Movie 6.

Description: Neural tube closure is initiated at two closure points in the forebrain. Time lapse imaging of embryo mosaically-expressing mGFP. The Green and brightfield channels are overlaid to reveal the shape of the neural folds and neural groove. Annotations: red asterisk: apex of the arch shaped neural folds; white dotted line: contour of the eye-shaped opening whose corners are defined by closure points 1 and 2, double headed arrow: width of the neural plate, which decreases over time, white arrows: closure points 1 and 2, respectively.

File name: Supplemental Movie 7.

Description: Neural fold cells extend filopodial protrusions across the midline. Time lapse imaging of embryo ubiquitously expressing mGFP. Cells (cyan and magenta) originating from contralateral sides of the ANP extend medially oriented filopodia and transiently interdigitate across the midline (yellow dashed line).
