# Supplementary material for: Hallmarks of primary neurulation are conserved in the zebrafish forebrain
Source: Commun Biol. 2021 Jan 29;4:147. doi: 10.1038/s42003-021-01655-8 (PMC7846805; doi:10.1038/s42003-021-01655-8)
Supplement: Supplementary file 10 — Reporting Summary [file 42003_2021_1655_MOESM10_ESM.pdf]

natureresearch

Corresponding author(s): Rachel Brewster

Last updated by author(s): Dec 17, 2020

Reporting Summary

Nature Research wishes to improve the reproducibility of the work that we publish. This form provides structure for consistency and transparency in reporting. For further information on Nature Research policies, see Authors & Referees and the Editorial Policy Checklist .

Statistics

For all statistical analyses, confirm that the following items are present in the figure legend, table legend, main text, or Methods section.

n/a	Confirmed	
☐	☒	The exact sample size (n) for each experimental group/condition, given as a discrete number and unit of measurement
☐	☒	A statement on whether measurements were taken from distinct samples or whether the same sample was measured repeatedly
☐	☒	The statistical test(s) used AND whether they are one- or two-sided<br>*Only common tests should be described solely by name; describe more complex techniques in the Methods section.*
☒	☐	A description of all covariates tested
☒	☐	A description of any assumptions or corrections, such as tests of normality and adjustment for multiple comparisons
☐	☒	A full description of the statistical parameters including central tendency (e.g. means) or other basic estimates (e.g. regression coefficient) AND variation (e.g. standard deviation) or associated estimates of uncertainty (e.g. confidence intervals)
☐	☒	For null hypothesis testing, the test statistic (e.g. F, t, r) with confidence intervals, effect sizes, degrees of freedom and P value noted<br>*Give P values as exact values whenever suitable.*
☒	☐	For Bayesian analysis, information on the choice of priors and Markov chain Monte Carlo settings
☒	☐	For hierarchical and complex designs, identification of the appropriate level for tests and full reporting of outcomes
☒	☐	Estimates of effect sizes (e.g. Cohen's d, Pearson's r), indicating how they were calculated

Our web collection on statistics for biologists contains articles on many of the points above.

Software and code

Policy information about availability of computer code

Data collection	All raw data was collected using ImageJ software.
Data analysis	Graphs were generated using the python Seaborn package with the following functions: seaborn.boxplot(), seaborn.lmplot(), seaborn.violinplot(), seaborn.lineplot(); or in R (version 3.6.0) with ggplot2 (version 3.3.0) using the functions geom_point() and geom_smooth(method = 'lm')

For manuscripts utilizing custom algorithms or software that are central to the research but not yet described in published literature, software must be made available to editors/reviewers. We strongly encourage code deposition in a community repository (e.g. GitHub). See the Nature Research guidelines for submitting code & software for further information.

Data

Policy information about availability of data

All manuscripts must include a data availability statement. This statement should provide the following information, where applicable:

- Accession codes, unique identifiers, or web links for publicly available datasets
- A list of figures that have associated raw data
- A description of any restrictions on data availability

The authors declare that all data supporting the findings of this study are available within the article and its supplementary information files or from the corresponding author upon reasonable request.

Field-specific reporting

Please select the one below that is the best fit for your research. If you are not sure, read the appropriate sections before making your selection.

Life sciences study design

All studies must disclose on these points even when the disclosure is negative.

Sample size	- Experimental procedures involving blebbistatin and myosin morpholino: sample sizes were chosen based on research articles using similar procedures (measurements of apical constriction) in the zebrafish embryos (e.g. PMID: 31097745).<br>- Measurements of neural fold convergence, cell shape and dynamic cell behaviors using WT embryos: sample sizes were also estimated based on research articles using similar procedures in the zebrafish embryos (e.g. PMID: 31097745, PMID: 31399534).
Data exclusions	No data was excluded.
Replication	All experiments were repeated multiple times (at least 3) and the results were found to be reproducible.
Randomization	Allocation of embryos to different experimental groups was randomized.
Blinding	Blinding was not relevant to this study as the bulk of the data collected was qualitative in nature (images of embryos/cells). With respect to quantitative analyses, data collection was performed using image processing software (ImageJ). Furthermoore, all embryos per experimental group were analyzed.

Reporting for specific materials, systems and methods

We require information from authors about some types of materials, experimental systems and methods used in many studies. Here, indicate whether each material, system or method listed is relevant to your study. If you are not sure if a list item applies to your research, read the appropriate section before selecting a response.

Materials & experimental systems

n/a	Involved in the study	
☐	☒	Antibodies
☒	☐	Eukaryotic cell lines
☒	☐	Palaeontology
☐	☒	Animals and other organisms
☒	☐	Human research participants
☒	☐	Clinical data

Methods

n/a	Involved in the study	
☒	☐	ChIP-seq
☒	☐	Flow cytometry
☒	☐	MRI-based neuroimaging

Antibodies

Antibodies used	Rabbit anti-GFP at 1:1000 (Invitrogen, A11122), Rabbit anti-Sox3c at 1:2000 (Gift from Michael Klymkowsky), Rabbit anti-P-myosin light chain at 1:50 (Cell Signaling Technology, #3671S), Mouse anti-p63 at 1:200 (Santa Cruz BioTechnology, SC-8431 no longer in production), Rabbit anti-N-cadherin at 1:50 (Abcam, ab211116) and Mouse anti-ZO-1 antibody at 1:100 (Invitrogen, 33-9100)
Validation	Anti-GFP: validation info available on manufacturer's website. Over 300 publications, including Stanic et al. 2018 (Expression of RPRM/rprm in the Olfactory System of Embryonic Zebrafish (Danio rerio)).<br>Anti-Sox3c was a gift from Michael Klymkowsky and has been used in many publications, including Zhang et al 2003 (The β-catenin/VegT-regulated early zygotic gene Xnr5 is a direct target of SOX3 regulation) and Harrington et al. 2007 (Cadherin-mediated adhesion regulates posterior body formation).<br>Anti-P-myosin: validation info available on seller's website. Over 250 publications, including Cao et al. 2017 (Tension Creates an Endoreplication Wavefront that Leads Regeneration of Epicardial Tissue).<br>Anti-p63: antibody discontinued, info availbale on http://datasheets.scbt.com/sc-8431.pdf.<br>Anti-N-cadherin: validation information available on seller website, zebrafish antibody<br>Anti-ZO-1: alidation information available on seller website. Over 700 publications, including Hehr et al. 2018 (Polarity and Morphogenesis of the Eye Epithelium Requires the Adhesion Junction Associated Adaptor Protein Traf4).

Animals and other organisms

Policy information about studies involving animals; ARRIVE guidelines recommended for reporting animal research

Laboratory animals	Species: Danio rerio<br>Strain: AB<br>Sex: embryos were not sexed, not possible<br>Age: developmental stages 2-10 somites were used, approximately 10-14 hours post fertilization

Wild animals	N/A
Field-collected samples	N/A
Ethics oversight	The UMBC IACUC committee

Note that full information on the approval of the study protocol must also be provided in the manuscript.
